# Efficacy of CRP in combination with D-dimer in predicting adverse postoperative outcomes of patients with acute Stanford type A aortic dissection

**DOI:** 10.1186/s13019-022-01818-6

**Published:** 2022-04-11

**Authors:** Zhiwei Tang, Hong Liu, Yongfeng Shao

**Affiliations:** Department of Cardiovascular Surgery, The First Affiliated Hospital of Nanjing Medical University, Nanjing, 210029 China

**Keywords:** CRP, D-dimer, In-hospital adverse outcomes, Aortic dissection, Predictive efficacy

## Abstract

**Purpose:**

The present study evaluated the efficacy of C-reactive protein (CRP) and D-dimer and the combination of them as prognostic indicators for patients with acute type A aortic dissection (ATAAD).

**Methods:**

This is a retrospective cohort study. From January 2019 to December 2021, patients with ATAAD admitted to the emergency medicine center of our hospital within 24 h after symptoms (chest pain, back pain, abdominal pain and so on) onset were enrolled in our study. Serum concentration of CRP and D-dimer were measured during hospitalization. Logistic regression was used to evaluate the association between these two biomarkers and in-hospital adverse outcomes (IAO) by adjusting confounding factors. Predictive efficacy was assessed by area under the curve (AUC) of receiver operating characteristic curve.

**Results:**

A total of 199 patients with ATAAD were finally enrolled. They were categorized as Non-IAO group (n = 146) and IAO group (n = 53) according to postoperative outcomes. After controlling for potentially confounding variables, we found categorized variables that admission CRP > 54.28 mg/L, admission D-dimer > 8.45 mg/L and peak D-dimer > 24.89 mg/L were independent predictors of in-hospital adverse outcomes. Multiple Logistic regression analysis revealed that the odd ratios were 2.9 for admission D-dimer > 8.45 [95% Confidence Interval (CI) 1.11–7.5, *p* = 0.03], 4.9 for admission CRP > 54.28 (95% CI 1.6–14.9, *p* = 0.005) and 5.7 for peak D-dimer > 24.89 (95% CI 2.49–13, *p* < 0.001). The predictive accuracy of the combination of three categorized variables (AUC: 0.867, 95% CI 0.813–0.921, *p* < 0.001) was superior to that of any other one alone.

**Conclusion:**

Admission D-dimer > 8.45 mg/L, peak D-dimer > 24.89 mg/L and admission CRP > 54.28 mg/L are independent predictors of in-hospital adverse outcomes in patients with ATAAD. Combination of these three markers will improve the predictive efficacy.

## Introduction

Acute Stanford type A aortic dissection (ATAAD) is a catastrophic cardiovascular emergency in adults, associated with high morbidity and mortality [1]. Inflammation and thrombosis are two main mechanisms that contribute to the pathogenesis of ATAAD [2]. Based on that, inflammatory and thrombotic biomarkers associated with in-hospital adverse outcomes are emerging [3]. Whether these inflammatory and thrombotic biomarkers as risk-prediction tools for the short-term results remains controversial. Perfect predictive methods can help the surgeon identify patients at higher risk of AD and thus providing appropriate and prompt medical intervention.

In recent years, CRP and D-dimer are proven to have prognostic significance in various cardiovascular diseases [4, 5]. Inflammation and thrombosis through a cascade of these markers (such as CRP and D-dimer) result in the onset of aortic dissection along with subsequent aortic rupture [1, 6]. It is important to choose the appropriate detection time window for these markers because of their exhibition in different time course of their changes in the acute phase [7, 8].

Although the individual prognostic ability of CRP, and D-dimer in ATAAD has been studied extensively, few studies have taken CRP and D-dimer exhibiting different time courses of their changes into consideration and investigated their combined efficacy of predicting in-hospital adverse outcomes. In the present study, we aimed at evaluating the efficacy of these two biomarkers obtained from serum tests alone and the combination of them as prognostic indicators for patients with ATAAD.

## Materials and methods

### Study cohort

From January 2019 to December 2021, patients diagnosed with ATAAD who were admitted to the emergency center of the First Affiliated Hospital of Nanjing Medical University were enrolled in our study. The study was approved by the ethical committee of the First Affiliated Hospital of Nanjing Medical University. The diagnosis of ATAAD was mainly confirmed by computed tomographic angiography (CTA). Patients with ATAAD within 24 h after symptom onset were enrolled in our study. Exclusion criteria were: (1) death due to aortic dissection rupture (2) symptoms lasting more than 24 h (3) history of chronic liver or kidney diseases.

### Study design

C-reactive protein and D-dimer were measured during admission and after surgery. We analyzed the association of admission C-reactive protein, admission D-dimer, and peak D-dimer with adverse outcomes after aortic surgery. Those biomarkers above were also presented as categorical variables according to optimal cutoff value in predicting in-hospital adverse outcomes with high sensitivity and specificity in the ROC curves.

### Definitions

In-hospital adverse outcomes were defined as Stage 3 acute kidney injury, stroke and mortality. Symptoms of patients with ATAAD were chest pain, back pain, abdominal pain, head or neck pain, severe or worst ever pain, abrupt onset of pain and syncope. AKI was ascertained and categorized according to the kidney disease: Improving Global Outcomes (KDIGO) Clinical Practice Guidelines. Those with ≥ 3.0-fold rise of serum creatinine during 10 days after surgery or ≥ 4 mg/dL (353.6 μmol/L) increase in 48 h were diagnosed as Stage 3 AKI [9]. Postoperative stroke was diagnosed by neuroimaging such as a CT or MRI head scan.

### Data collection

We collected consistent data for each patient from the medical records. Based on detailed literature reviews and clinical evidence, we selected all candidate predictors within the confines of data availability. Baseline characteristics data included continuous and categorized sex, age, and hypertension. The clinical profiles included serum concentration of leukocyte counts, neutrophil counts, lymphocyte counts, monocyte counts, platelet counts, hemoglobin, ALT, AST, Cr, BUN, CRP, and D-dimer. In-hospital adverse outcomes of enrolled patients including survival, stage 3 acute kidney injury, stroke and mortality were recorded.

### Statistical analysis

All statistical analyses were performed by the Statistical Package for the Social Sciences (SPSS) 23.0. Variables were expressed as frequencies (percentages) for categorical variables and medians (interquartile ranges [IQRs]) for continuous variables. Differences between groups were assessed using the t test or the Mann–Whitney U test for continuous variables and the χ^2^ test or Fisher exact test for categorical variables. Logistic regression analysis was used to investigate the association of these two biomarkers with adverse outcomes, after adjustment for confounding factors. The covariates considered were age, MLR, platelet, AST, Cr, peak levels of D-dimer during hospitalization, admission CRP and D-dimer. We used last carry-over method to assess the sensitivity of results to missing values. All variables that were to be included in the regression analysis were used in the imputation process. Receiver operating characteristic (ROC) analysis was performed to determine the cut-off value for CRP and D-dimer in predicting in-hospital adverse outcomes with high sensitivity and specificity. *p* < 0.05 was considered statistically significant (two-sided).

## Results

### Baseline characteristics of participants

**Fig. 1:**
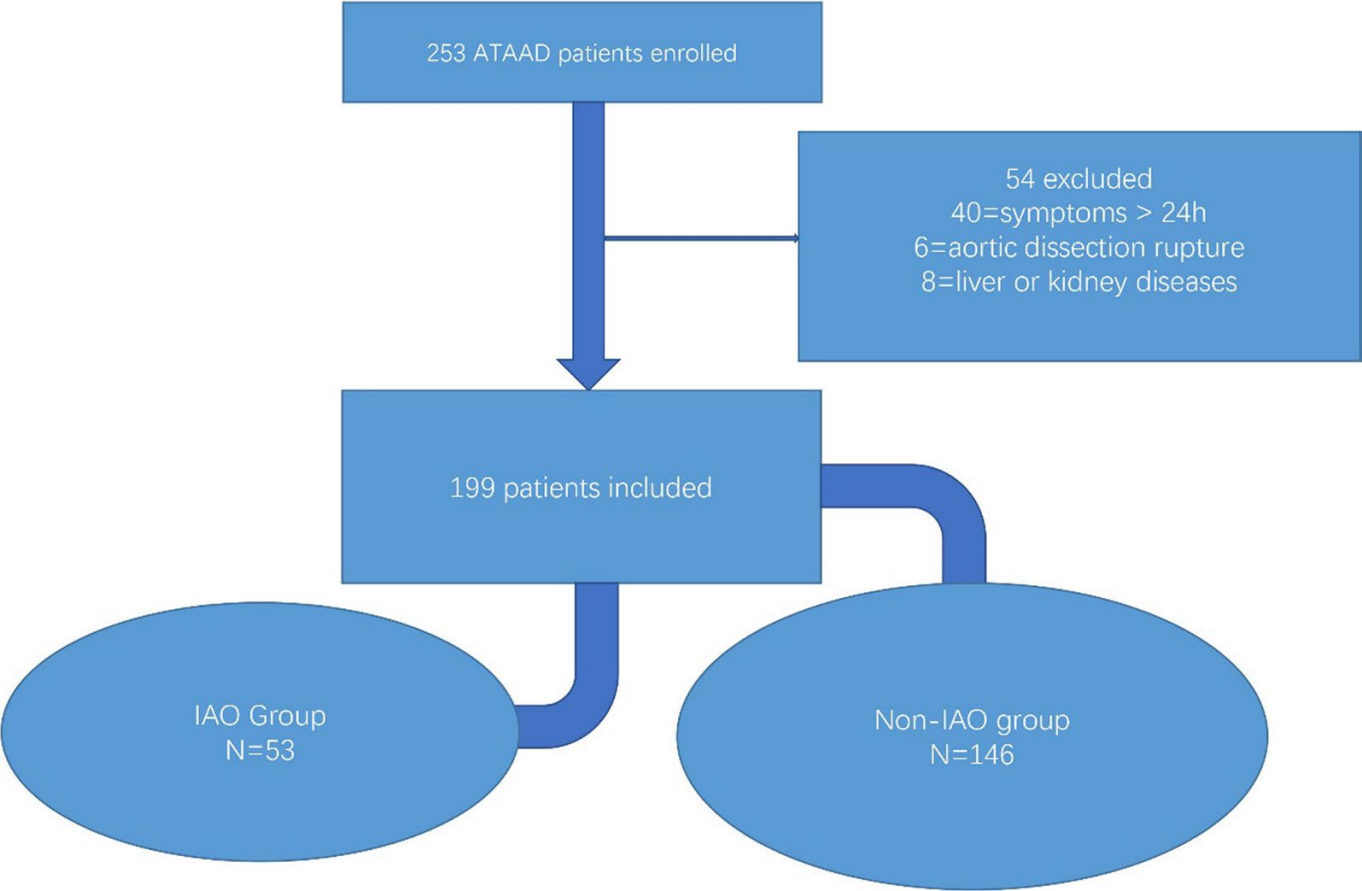
Patient Selection. ATAAD: acute Stanford type A aortic dissection. *IAO* In-hospital adverse outcomes

253 Patients diagnosed with ATAAD were identified in the present study between January 1, 2019 and December 31, 2021. Of these patients, 40 were excluded for symptoms lasting more than 24 h. 6 were excluded because of aortic dissection rupture. 8 patients were excluded because of history of chronic liver or kidney diseases. Finally, a total of 199 patients with ATAAD were studied. There were 149 male and 50 female patients. Baseline characteristics of two cohorts grouped by postoperative outcomes were listed in Table 1. Admission D-dimer was 3.13(1.38, 7.96) in the non-IAO group and 9.47 (3.35, 22) in the IAO group. Admission C-reactive protein was 7.45(5, 20.6) in the non-IAO group and 9.2(5.85, 55) in the IAO group (*p* < 0.01). Patients in the IAO group (57.2 ± 10.6) were significantly older than those were in the non-IAO group (53.2 ± 13.1). Patients in the non-IAO group (0.83(0.52, 1.06)) had significantly higher MLR than those did in the IAO group (0.66(0.46, 0.94)). Patients in the non-IAO group had significantly lower values of AST than those did in the IAO group. Patients in the non-IAO group (174.5(133.75, 213.00)) had significantly higher platelets than those did in the IAO group (146.00(114, 174)). For creatinine, the levels of patients in the IAO group were significantly higher than those were in the non-IAO group (Fig. 1).

**Table 1:** Baseline characteristics of patients grouped by postoperative outcomes

Variables	Non-IAO group (n = 146)	IAO group (n = 53)	*p* Value
Age(years)	53.2 ± 13.1	57.2 ± 10.6	0.05
Male gender (n, %)	113(77.4)	36(67.9)	0.17
Hypertension, n (%)	130(87.2)	44(83)	0.44
*Admission data*			
WBC (10^9^/L) median (IQR)	12(9.53, 14.5)	13(10.9, 14.9)	0.096
NLR	11(6.4, 15.7)	10.8(5.6, 17.5)	0.58
MLR	0.83(0.52, 1.06)	0.66(0.46, 0.94)	0.03
Hemoglobin	138.5(127, 150)	134(119, 145)	0.10
Platelet (10^9^/L)	174.5(133.75, 213.00)	146.00(114, 174)	< 0.01
D-dimer (mg/L)	3.13(1.38, 7.96)	9.47 (3.35, 22)	< 0.01
CRP (mg/L)	7.45(5, 20.6)	9.2(5.85, 55)	0.029
ALT	31(23.7, 43.75)	33(25.25, 44.7)	0.43
AST	27.1(21.48, 36.95)	31.8(25.2, 45)	0.017
Creatinine	72.95(56.95, 94.73)	84.8(63.95, 105.45)	0.043
BUN	6.5(5.5, 8.2)	7.6(5.5, 9.7)	0.075
*Postoperative data*			
Peak D-dimer	12.7(8.9, 21.9)	34.6(17.3, 40)	< 0.01

### ROC curves of biomarkers for in-hospital adverse outcomes

**Fig. 2:**
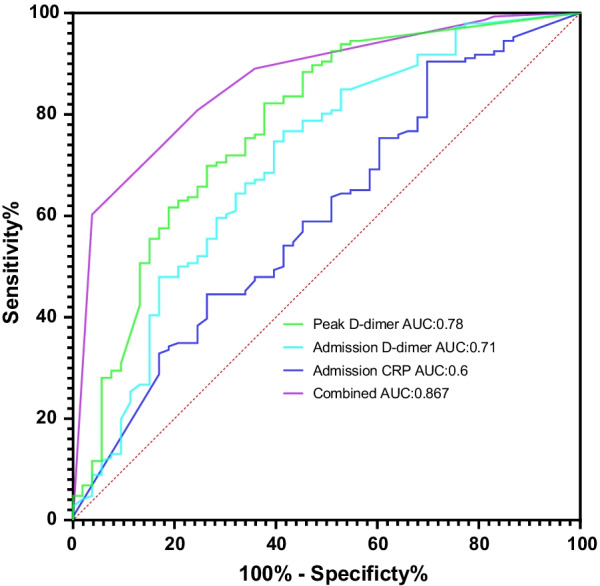
Receiver operating characteristic (ROC) curves of combination of categorized admission D-dimer, admission CRP and peak D-dimer for in-hospital adverse outcomes

ROC curves for in-hospital adverse outcomes of admission D-dimer, admission CRP and peak D-dimer were shown in Fig. 2. With optimal cutoff value of 54.28, CRP exhibited sensitivity of 30.2%, specificity of 90.4%. With optimal cutoff value of 8.45, D-dimer exhibited sensitivity of 58.5%, specificity of 76.7%. Peak D-dimer exhibited sensitivity of 62.3%, specificity of 82.2% with optimal cutoff point of 24.89.

### Multivariate logistic regression analysis of predictors for in-hospital adverse outcomes

It was shown in the Table 2 that after controlling for potentially relevant confounding variables, admission C-reactive protein > 54.28 mg/L, admission D-dimer > 8.45 mg/L and peak D-dimer > 24.89 mg/L were independent predictors for in-hospital mortality in multivariate logistic regression analysis (odds ratio, 4.9; 95% CI 1.6–14.9, *p* = 0.005; odds ratio, 2.9; 95% Confidence Interval (CI) 1.11–7.5, *p* = 0.03, OR, 5.7; 95% CI 2.49–13, *p* < 0.001, respectively).

**Table 2:** Multivariate logistic regression analyses of the prognostic factors for in-hospital adverse outcomes in patients with TAAD

Variables	OR	95%CI	*p* Value
*Admission data*			
D-dimer > 8.45 (mg/L)	2.9	1.11–7.5	0.03*
C-reactive protein > 54.28 (mg/L)	4.9	1.6–14.9	0.005*
*Postoperative data*			
Peak D-dimer > 24.89 (mg/L)	5.7	2.49–13	< 0.001*

*P < 0.05, Adjusted for age, MLR, platelet, AST, and Cr. Other covariates had no significance

### Combination of biomarkers to predict in-hospital adverse outcomes

To get a better prediction for in-hospital adverse outcomes, we combined these three categorized biomarkers (admission CRP, admission D-dimer and peak CRP) that could predict in-hospital adverse outcomes in multivariate regression models of each patient on a ROC. The AUC was 0.867 (95% CI 0.813–0.921, *p* < 0.001) (Fig. 2).

## Discussion

This study showed that CRP and D-dimer are useful predictors of in-hospital adverse outcomes in patients with ATAAD. Those who had admission CRP > 54.28 mg/L, admission D-dimer > 8.45 mg/L and peak D-dimer > 24.89 mg/L within 24 h after symptom onset were prone to in-hospital adverse outcomes. Moreover, combination of these three biomarkers (admission CRP, admission D-dimer and peak D-dimer) were more predictive than any marker alone, which was the strongest (AUC:0.867). The calculation of these two inexpensive biomarkers is easy to get without paying more either for the individual or the medical system, which increases the potential value.

Biomarkers in the acute inflammatory and thrombotic response were associated with the prognosis of ATAAD [10–14]. The inflammation within the wall damages the aorta, making it easily enlarge and vulnerable to rupture. The coagulation system is activated by extensive inflammation and the reverse is also true via crosstalk [15]. Attention have been focused on the prognostic value of individual indicators such as MLR and NLR for ATAAD in previous studies [3, 16], which is unable to obtain an ideal prediction efficacy. New research demonstrates that ATAAD results from a combination of inflammation and thrombosis [2]. CRP plays an important role in the inflammatory state of ATAAD, while platelets and D-dimer reflect the thrombotic state in ATAAD [17, 18]. Although previous studies confirmed that CRP and D-dimer exhibited different time course of their changes in the acute phase [8, 9], time from the symptom onset of patients in the present study is within 24 h, which attenuates the impact. Hence, these two biomarkers namely CRP and D-dimer that integrate multiple pathways of inflammatory and thrombotic processes may provide a more predictive assessment of the prognosis of ATAAD patients. We have also demonstrated that the combination of inflammatory biomarkers (CRP) and thrombotic marker (D-dimer) is a strong predictor of in-hospital adverse outcomes than either of these two biomarkers alone. Besides, the two biomarkers were obtained from serum samples, which were readily available and greatly significant in terms of economy for poor areas, compared to other laboratory tests for ATAAD.

CRP is a sensitive and non-specific inflammatory biomarker, readily available and relatively inexpensive in routine clinical practice [19]. CRP which is mainly synthesized by hepatocytes driven by the stimulation of various cytokines associated with inflammation is upregulated in AAD [20]. Its plasma levels depend on inflammatory stages. CRP has been thought to be significantly linked to the occurrence and development of AD, suggesting its participation in the inflammatory pathways in AD [21, 22]. CRP values were significantly higher in patients who suffered in-hospital adverse outcomes compared with those who did not suffer, indicating that CRP was useful for prognostic stratification of patients with type A AD. In line with the results of previous studies, the present study further proved the value of admission CRP in predicting in-hospital adverse outcomes in ATAAD. We also found admission > 54.28 during hospitalization was also independent predictors of in-hospital adverse outcomes. The odd ratio was 4.9 for admission CRP > 54.28 (95% Confidence Interval (CI) 1.6–14.9, *p* = 0.005).

D-dimer represents a protein fragment produced by crosslinked fibrin detectable in plasma following thrombus fibrinolysis. D-dimer is majorly tested for the diagnosis and prognosis of pulmonary embolism, disseminated intravascular coagulation and aortic dissection. Levels of D-dimer not only are elevated in patients with pulmonary embolism, deep-vein thrombosis and disseminated intravascular coagulation but also increase in cancer, infections, elder age and surgery, resulting in high diagnostic sensitivity but low specificity as a result [23–26]. D-dimer has a longer half-life period and it is a meaningful biomarker in predicting in-hospital mortality. In the present study, admission D-dimer > 8.45 was associated with an odd ratio of 2.9 [95% Confidence Interval (CI) 1.11–7.5, *p* = 0.03] in predicting in-hospital adverse outcomes demonstrating that D-dimer was an independent risk factor for in-hospital adverse outcomes in ATAAD. Moreover, we also found peak D-dimer > 24.89 mg/L during hospitalization was also independent predictors of in-hospital adverse outcomes. The odd ratio was 5.7 for peak D-dimer > 24.89 [95% Confidence Interval (CI) 2.49–13, *p* < 0.001].

Previous studies demonstrated that AD is associated with platelet activation and adhesion to the damaged vessel walls, which may form a thrombosis in the false lumen [27]. Platelet dysfunction which marks serious thrombotic burden has been observed in patients with ATAAD [13]. The excessive consumption of platelets after thrombosis may make the damaged aortic wall prone to rupture, which increases mortality [12]. Huang et al. [14] found that admission levels of platelet count < 119 × 109/L was associated with an odds ratio of 3.90 (95% CI 1.67–9.09) for in-hospital mortality. However, in the present study, platelet counts were not significantly associated with IAO in the multivariate logistic regression analyses.

Chen et al. demonstrated that individual biomarker like neutrophil to lymphocyte ratio and monocyte to lymphocyte ratio was unable to predict in-hospital mortality in patients with type A AAD [3]. Liu Jun et al. observed that fibrinogen was a powerful predictor of mortality in patients with ATAAD [4]. There is no statistical significance of biomarkers discussed above in the present study.

The limitations of the present study were as follows: (1) this is a retrospective study and the outcome may be affected by many confounding factors. Large multicentric randomized controlled trials are needed in the future. (2) it tends to exaggerate the predictive value based on the existing data to explore the appropriate predictive cut point on ROC curves. We need to divide the study population into test queue and validation queue, so we can get the cut point in test queue and evaluate its predictive efficacy in validation queue.

## Conclusion

Admission D-dimer > 8.45 mg/L, peak D-dimer > 24.89 mg/L and admission CRP > 54.28 mg/L were independent predictors of in-hospital adverse outcomes of patients with ATAAD within 24 h after symptoms onset. Combination of these readily available markers would improve the efficacy.

## Data Availability

Please contact author for data requests.

## Abbreviations

NLR: NEUTROPHIL to lymphocyte ratio
MLR: Monocyte to lymphocyte ratio
ROC: Receiver operating characteristic
OR: Odd ratio
AUC: Area under the curve
CTA: Computed tomography angiography
ATAAD: Acute Stanford type A aortic dissection
CRP: C-reactive protein
IAO: In-hospital adverse outcomes
Cr: Creatinine
ALT: Alaninetransaminase
AST: Aspartate amino transferase
BUN: Blood urea nitrogen
